# Compatibility of Post-Kidney Transplant Immunosuppression Therapy with Lactation

**DOI:** 10.3390/jcm14072364

**Published:** 2025-03-29

**Authors:** Gema Gomez-Casado, Juana Alonso-Titos, Ernesto Gonzalez-Mesa, Almudena Ortega-Gomez

**Affiliations:** 1 Instituto de Investigación Biomédica de Málaga—IBIMA Plataforma BIONAND, University of Malaga, 29010 Malaga, Spain; gema.gomez@ibima.eu (G.G.-C.); juana12041988@hotmail.com (J.A.-T.); egonzalezmesa@uma.es (E.G.-M.); 2 Department of Endocrinology and Nutrition, Virgen de la Victoria University Hospital, 29010 Málaga, Spain; 3 Nephrology Department, Regional University Hospital of Malaga, RICORS2040 (RD21/0005/0012-RD24/004/0026), 29010 Malaga, Spain; 4 Department of Surgical Specialties, Biochemistry and Immunology, Faculty of Medicine, University of Malaga, 29010 Malaga, Spain; 5 Department of Obstetrics and Gynecology Service, Regional University Hospital of Malaga, 29010 Malaga, Spain; 6 CIBER Fisiopatologia Obesidad y Nutricion (CIBEROBN), Instituto de Salud Carlos III, 28029 Madrid, Spain

**Keywords:** kidney transplant, immunosuppression therapy, lactation, breastfeeding

## Abstract

Breastfeeding after kidney transplantation remains a complex and underexplored topic, primarily due to concerns regarding the safety of immunosuppressive therapies during lactation. Individuals who have received kidney transplants face a higher likelihood of delivering preterm infants and giving birth to babies with a low birth weight when compared with the general population. In this context, breastfeeding is increasingly important because of its advantages for preterm infants. Despite the well-established benefits of breastfeeding for both the mother and infant, the traditional recommendation has been to avoid nursing due to potential drug transmission through breast milk. However, emerging evidence suggests that certain immunosuppressants may be compatible with breastfeeding, challenging long-standing clinical guidelines. In this review, we examine the current literature on the pharmacokinetics, safety profiles, and clinical outcomes associated with key immunosuppressive agents, including cyclosporine, tacrolimus, everolimus, azathioprine, corticosteroids, and belatacept. Our work highlights that all published reports to date on the studied treatments indicate that the amount of the drug reaching breast milk is considered safe for the child’s health. These conclusions, however, are derived from very short-term measurements and small numbers of patients. Therefore, we emphasize the need to design structured prospective studies to assess safety in the medium and long term. Our review aims to equip clinicians with the most up-to-date evidence on this topic, enabling them to make informed decisions regarding the compatibility of post-kidney transplant treatments with breastfeeding.

## 1. Introduction

Women within the reproductive age range (18–49 years) who suffer from end-stage renal disease (ESRD) experience fertility rates that are almost 10 times lower compared to their healthy peers. According to the National Transplant Registry (NTPR), the live birth rate among recipients is between 71% and 79%, while the UK Transplant Registry reports a live birth rate of 79% [1]. The live birth rate among allograft recipients is similar to that of the general population. The occurrence of preterm deliveries has been noted to reach between 40% and 60%, in contrast to the 5% to 15% seen in the general population, primarily resulting from complications involving the mother or fetus rather than spontaneous preterm labor [1]. Furthermore, they have a higher prevalence of low birth weight (42–46%) and intrauterine growth restriction (30–50%) [2,3]. Additionally, the incidence of miscarriage ranges from 11% to 26%, in contrast to the 8% to 9% observed in the general population [1,4]. The effects of a low and extremely low birth weight on the fetus are significant, encompassing a range of neurological, endocrine, cardiac, and renal issues [5]. Research on the developmental outcomes in children born to transplant recipients is scarce. Data from the National Transplantation Pregnancy Registry (NTPR), based in the US, are limited but indicate that developmental delays have been observed in as many as 26% of children older than 5 years [1]. In situations of infertility, transplanted women often seek out assisted reproductive methods, with in vitro fertilization being the most common reproductive technique used. Case reports and population-based retrospective studies document favorable outcomes of pregnancies in transplanted mothers following induction medication treatment or in vitro fertilization [6].

Breastfeeding is the most effective way to support a child’s growth and development. As a result, the World Health Organization recommends exclusive breastfeeding for the first 6 months and continued complementary breastfeeding for at least 3 years [7]. Children who are breastfed for more extended durations experience reduced rates of infectious diseases and deaths when compared to those breastfed for shorter times or not breastfed at all [8]. This difference persists into later life stages. Growing evidence suggests that breastfeeding also offers a safeguard against the risks of future overweight/obesity and diabetes [9,10]. For preterm infants, breastfeeding is crucial to their care and provides significant benefits for their growth and development. It helps reduce the risk of conditions such as necrotizing enterocolitis and late-onset sepsis [11,12], bronchopulmonary dysplasia [13], retinopathy of prematurity [14], and the likelihood of being readmitted to the hospital during the first year of life [15,16]. Additionally, it is linked to better neurodevelopmental results and enhanced cardiac performance [17]. Given the elevated rates of preterm births in pregnancies following kidney transplantation, breastfeeding is crucial for offering optimal support to infants born to mothers who have undergone transplantation.

## 2. Immunosuppression After Kidney Transplant

Graft damage and loss due to allograft rejection is a significant issue, with 7.0% of adult kidney transplant recipients experiencing acute rejection by 1 year [18]. There are two main types of rejection: T-cell-mediated rejection (TCMR) and antibody-mediated rejection (AMR). TCMR happens when donor-reactive CD4 and/or CD8 T cells infiltrate the allograft, causing inflammation and tissue damage [19]. On the other hand, AMR occurs when donor-specific antibodies bind to the allograft’s endothelium, triggering the complement system and attracting leukocytes, which lead to graft damage [20]. The primary goal of immunosuppression is to enhance the longevity of the transplanted organ by avoiding rejection while reducing the side effects associated with treatment. After a transplant, immunosuppression consists of two stages: induction immunosuppression, given around the time of surgery to avert immediate rejection, and maintenance immunosuppression, provided on a continuous basis to support prolonged graft survival [21].

The administration of these immunosuppressants, which tend to be more effective with fewer adverse effects, has notably reduced both the mortality and morbidity rates. Immunosuppressive therapies include various agents, such as calcineurin inhibitors (e.g., cyclosporine-A and tacrolimus), antimetabolites (e.g., azathioprine), mTOR inhibitors (e.g., sirolimus and everolimus), and steroids. While these immunosuppressive drugs can lead to numerous side effects, such as hypertension, infections, and increased lipid levels, they are essential for preventing organ rejection. This highlights the necessity for personalized medication strategies [22]. Newer medications are key to enable more personalized treatment options. Selecting an appropriate immunosuppressive regimen should be personalized for each patient, considering the pharmacological properties of the drugs, their side effect profiles, potential interactions with other medications, as well as the patient’s existing health conditions, risk of rejection, and current medication regimen. In this regard, evaluating how the treatment aligns with pregnancy and breastfeeding for individuals of reproductive age is essential.

## 3. Post-Kidney Transplant Immunosuppressive Treatments

Immunosuppression is generally accomplished by limiting the activity of lymphocytes. Over the decades, several treatment strategies have been employed to achieve this goal, including the use of calcineurin inhibitors, antiproliferative agents, mTOR inhibitors, and corticosteroids. These therapies, often used in combination, have evolved to enhance graft survival while minimizing adverse effects and the risk of infection. Continuous advancements in immunosuppressive regimens aim to refine this balance, improving long-term outcomes for kidney transplant recipients.

The arrival of calcineurin inhibitors (CNIs), specifically cyclosporine and tacrolimus, resulted in a reduction of rejection rates and enhanced short-term outcomes for both patients and grafts. However, prolonged use of these medications has been associated with kidney graft dysfunction, hypertension, hyperlipidemia, and diabetes mellitus. Sirolimus and everolimus, which are inhibitors of the mammalian target of rapamycin (mTOR), demonstrate effectiveness comparable to that of cyclosporine but come with side effects like delayed wound healing and hyperlipidemia [23,24,25,26]. Azathioprine is a pro-drug of 6-mercaptopurine (6-MP), a purine antagonist that inhibits leukocyte proliferation by interfering with nucleotide synthesis. Cytotoxic thioguanine nucleotides decrease the production of purine nucleotides via the de novo pathway by inhibiting amidotransferase enzymes and the interconversion of purine ribonucleotides. This mechanism is believed to cause the impact of azathioprine on the proliferation of leukocytes [27]. The corticosteroids utilized in transplantation medicine are primarily glucocorticoids, which are employed for their immune-modulating effects on the host’s immune system to help reduce and minimize rejection. Glucocorticoids influence cells by attaching to the glucocorticoid receptor. The complex formed by the activated glucocorticoid receptor and glucocorticoid boosts the production of anti-inflammatory proteins in the nucleus (a process known as transactivation) and inhibits the synthesis of pro-inflammatory proteins in the cytosol by preventing the translocation of other transcription factors from the cytosol to the nucleus (transrepression) [28].

To mitigate the potential for nephrotoxicity associated with conventional therapies, newer treatments, including belatacept, a selective T-cell co-stimulation blocker, have been authorized in recent decades.

**Figure 1:**
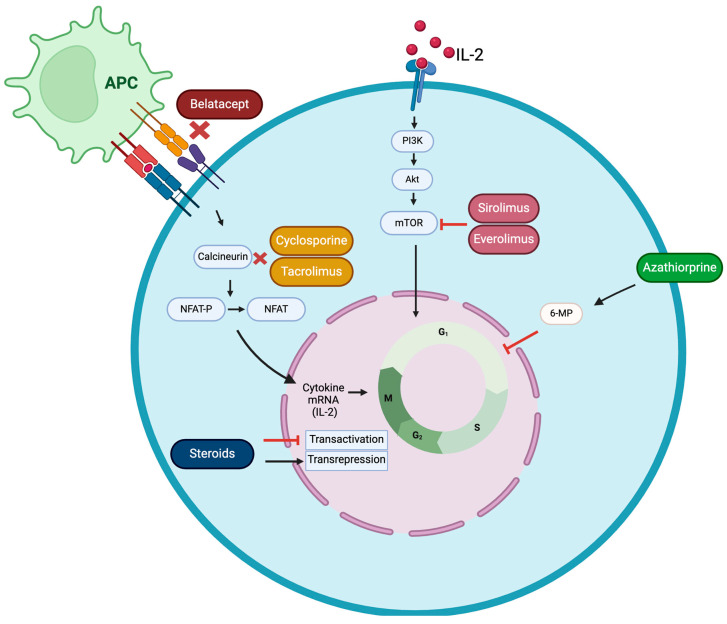
Mechanisms of action of most common immunosuppressive drugs: belatacept, cyclosporine, tacrolimus, sirolimus, everolimus, azathioprine, and steroids. APC: antigen-presenting cell.

The mechanisms of action of the mentioned immunosuppressive drugs can be found in Table 1 and Figure 1.

## 4. Immunosuppressive Drugs and Breastfeeding

Individuals of reproductive age who have received transplants need guidance regarding the possible teratogenic effects associated with immunosuppressive therapy. Since pregnant women were traditionally excluded from trials involving immunosuppressants, safety information primarily comes from animal research and epidemiological studies derived from transplant pregnancy registries and specific case reports [29]. While acknowledging the potential risk of contact with immunosuppressants, it is important to consider that an infant’s exposure to these substances through breastfeeding is less than that experienced in utero. The appropriateness of using immunosuppressive therapy during pregnancy has been examined in other sources, so it will not be addressed in this review in order to focus on breastfeeding [30,31,32,33]. Consequently, we explore the information available regarding the impact of maintenance immunosuppressive therapy in lactation.

To assess the exposure of children to medications taken by mothers through breast milk, several critical pharmacokinetic factors must be considered. On the one hand, it is important to understand how a medication transfers from the mother’s bloodstream into breast milk and the anticipated concentration of the medication at specific times after administration. On the other hand, the expected absorption of the medication in the infant’s gastrointestinal system and the amount of breast milk consumed are important factors. The transfer of medications from the maternal bloodstream to breast milk primarily occurs through passive diffusion. Similar to their transfer through the placenta, medications that are poorly protein bound, possess small molecular weights (less than 800 Dalton), and are highly lipophilic are more likely to be transferred into breast milk compared to larger and more hydrophilic substances [34].

The milk-to-plasma concentration ratio (M/P) of drugs is used to assess the amount of medication transferred to the breastfeeding infant. A M/P value of less than 1 indicates a low transfer of medication into breast milk. There is dependable information on M/P concentration ratios for only a limited number of drugs. The percentage of drug intake by infants through milk (adjusted for weight and time) is also utilized to examine how maternal drugs are distributed to infants. However, at this time, there is no suitable model available to forecast drug concentrations in human milk [34]. Likewise, a relative infant dose (RID), calculated as the weight-adjusted percentage of the maternal dose, which evaluates the projected infant dose based on the concentration of medication in breast milk and the volume ingested against the mother’s dosage, of less than 10% is regarded as safe. Caution is advised for drugs that are excreted in amounts ranging from 10% to 25% of the maternal dose, while those excreted in quantities exceeding 25% are generally deemed unacceptable [35].

The availability of data regarding the safety of immunosuppressive treatment after transplantation is severely limited by the traditional recommendation to avoid breastfeeding during this therapy [36,37,38]. However, in recent decades, new research has surfaced that demonstrates the compatibility of certain immunosuppressive medications following organ transplants. In the following sections, we will delve into the available evidence regarding the most commonly used drugs, analyzing their mechanism of action, safety profiles, and potential interactions in post-transplant care.

### 4.1. Calcineurin Inhibitors

Calcineurin inhibitors (CNIs) are an essential class of immunosuppressive drugs commonly used to treat a range of autoimmune conditions. In addition to these applications, CNIs are vital in preventing organ rejection by providing crucial immunosuppression in solid organ transplantation. The most commonly used CNIs in post-transplant immunosuppressive therapy are cyclosporine and tacrolimus (Table 1). These drugs work by binding with high specificity and affinity to specific cytoplasmic receptors, collectively known as immunophilins, such as cyclophilin and FK-binding proteins. By inhibiting calcineurin, CNIs block a key signaling pathway that leads to the transcription of interleukin-2 and other important cytokines, like IL-4, interferon-γ (IFN-γ), and tumor necrosis factor-α (TNF-α). This pathway involves NFAT, a DNA-binding protein crucial for interleukin-2 gene transcription. In unstimulated T cells, NFAT consists of a subunit (NFATp) that interacts with Fos and Jun proteins in the nucleus of activated T cells [39]. The blockage of this route disrupts the activation, proliferation, and differentiation of T lymphocytes, which are integral to the immune response, thereby reducing the risk of rejection in transplant recipients and controlling autoimmune activity in affected individuals [40] (Figure 1). The enzyme CYP3A4, which is responsible for metabolizing both CNIs, shows an increase in its expression within the neonatal liver after birth. This upregulation leads to a reduced concentration of these drugs in the bloodstream of infants shortly after delivery [41].

#### 4.1.1. Cyclosporine

In the majority of breastfed infants, cyclosporine is undetectable in the bloodstream. However, there have been instances where detectable blood levels were observed, even when both the milk concentration and the infant’s calculated dose were relatively low. Cyclosporine levels exhibit significant variability across different case reports and series assessed. This variation is likely due to discrepancies in sampling times across studies and is probably influenced by the fat content of the milk at the time of collection. When maternal cyclosporine blood levels are within the typical range, a fully breastfed infant usually receives no more than 2% of the mother’s weight-adjusted dose or the pediatric transplantation maintenance dose and often less than 1% [42]. There have been no reported cases of adverse effects on infants’ growth, development, or kidney function. As a result, guidelines from the United States and Europe, along with the National Transplantation Pregnancy Registry and other experts, deem the use of cyclosporine during breastfeeding to be acceptable [43,44]. However, it is important to note that comprehensive follow-up examinations may not have always been conducted or documented.

We have identified 14 reports, spanning from 1983 to 2022, that examine the transfer of cyclosporine into breast milk and its short-term effects on infants. The amount of cyclosporine detected in breast milk in these studies varied between 0.4 µg/mL and 564 µg/mL, and the maternal weight-adjusted dose ranged between 0.01% and 2.1%, far below the limit of 10% to be considered safe [35]. When reported, no clinical adverse effects were found in the infants. However, most of these studies are based on a single patient and are limited to short-term follow-up, with little consistency between articles.

In addition to case reports, studies conducted on animals have demonstrated the harmless effects of cyclosporin during lactation. One particular study found that although the levels of cyclosporin in mother rats were quite high, the cyclosporin levels in the pups from the 21-day treatment group were non-existent. Renal histomorphometric comparisons between the study pups and the control pups showed no significant differences in either age group. However, when examining renal function parameters, significant distinctions were observed between the study and control pups in the infancy category: pups in the 21-day treatment group exhibited a notably lower urine volume, proteinuria, FE (Na), and urinary NAG/creatinine ratio. Although the glomerular filtration rate was lower in the 21-day treatment group, this difference was not statistically significant, and there were no significant variations in serum creatinine levels [45]. A compilation of human studies testing the effect of cyclosporin on breastfeeding is detailed in Table 2.

#### 4.1.2. Tacrolimus

Tacrolimus, previously known as FK506, is a macrolide antibiotic that has immunosuppressive capabilities. While it is structurally different from cyclosporin, its mechanism of action is comparable. Tacrolimus exerts its primary effects by disrupting gene expression in target cells. It binds to an immunophilin called FK506 binding protein (FKBP), forming a complex that interferes with calcineurin phosphatase. This action inhibits calcium-dependent processes, including interleukin-2 gene transcription, the activation of nitric oxide synthase, cell degranulation, and apoptosis. Tacrolimus also amplifies the effects of glucocorticoids and progesterone by binding to FKBPs in the hormone receptor complex, preventing their degradation. The drug may increase the expression of the TGF-β gene, similar to cyclosporine. Tacrolimus inhibits T-cell proliferation in response to T-cell receptor ligation and appears to preferentially suppress type 1 T helper cells over type 2 T helper cells. B-cell growth and antibody production are indirectly affected by the suppression of T-cell-derived growth factors essential for these activities. Antigen presentation appears to remain unaffected. The molecular mechanisms influenced by tacrolimus are still being explored [39].

Twelve studies have been found on tacrolimus, its transfer to breast milk, and its effect on children. The amount of drug found in breast milk ranged between 0.5 μg/mL and 3.8 μg/mL, while the infant blood drug levels varied between 0.2 μg/L and 3.2 μg/L. In any case, the lowest maternal weight-adjusted dose value was 0.23%, and the highest was 0.5%, with both of them considered safe. The infants were reported to grow and develop normally, physically and neurologically, with no signs of metabolic disorders or significant infections. Although in this case there are several reports studying a group of patients ranging from 4 to 14, the results should be interpreted with caution due to the disparity between studies. Table 2 provides a summary of the studies examining the impact of tacrolimus on breastfeeding.

### 4.2. Thiopurine Drugs (Antimetabolites)

Thiopurine medications are purine antimetabolites commonly utilized in treating acute lymphoblastic leukemia, autoimmune conditions (such as Crohn’s disease and rheumatoid arthritis), and for patients who have undergone organ transplants. As pro-drugs that are inactive in their initial form, 6-mercaptopurine (6-MP), 6-thioguanine (6-TG), and azathioprine must undergo intracellular activation, which is facilitated by various enzymes, in order to produce their cytotoxic effects [46].

#### Azathioprine

Azathioprine is a pro-drug of 6-MP that was originally investigated as a chemotherapeutic drug for the treatment of leukemia [28]. 6-MP acts as a purine antagonist, hindering the proliferation of leukocytes by disrupting nucleotide synthesis (Figure 1). Azathioprine has a superior therapeutic index compared to 6-MP, which has resulted in azathioprine playing a more prominent role in the treatment options for managing systemic autoimmune diseases [47]. There have been rare reports of hyperprolactinemia and galactorrhea occurring with normal prolactin levels [48,49]. However, expert guidelines from North America and Europe, along with the US-based NTPR and other specialists, regard azathioprine as a suitable option for use while breastfeeding [50,51,52]. Thirteen studies have been identified reporting on azathioprine medication during breastfeeding, with a study group size between 1 and 34 patients. When reported, the levels of azathioprine or 6-MP measured in breast milk across these studies ranged from 1.2 to 50 µg/mL, being measured at different time points. High drug levels were undetected in the infant’s blood, and the calculated maternal weight-adjusted dose ranged between 0.05 and 1%, which is, in principle, considered safe. One infant was reported to have a borderline low neutrophil count but a normal white cell count. Otherwise, no adverse effects were encountered in the infants, who showed normal growth, normal mental and physical development, and no above-average infection incidence. In Table 2, a summary of reports on the impact of azathioprine on breastfeeding is presented.

**Table 2:** Reports on compatibility of most commonly used immunosuppressive drugs with breastfeeding.

Immunosuppressive Agent	Dose	Subject Group	Drug Levels in Breast Milk	Effects on the Infant	Year	Reference
Cyclosporine	450 mg/day during pregnancy	1	101, 109, and 263 μg/L in breastmilk on days 2, 3, and 4 postpartum	Not specified.	1983	[53]
	325 mg 2 h before the onset of labor	1	16 μg/L	Undetectable (<3 μg//L) cyclosporine blood levels.	1985	[54]
	225 mg/day	1	Not reported	Estimated intake of 6 μg/kg daily (0.01% maternal weight-adjusted dose); the infant remained healthy and normal.	1995	[55]
	3 mg/kg twice daily	1	596 μg/L 5 weeks postpartum; the infant would receive less than 0.1 mg/kg per day or no more than 1.7% of the maternal weight-adjusted dose	At 5 weeks of age, normal renal function and a blood cyclosporin concentration below 3 μg/L; estimated levels taken through milk (150 mL/Kg/day) < 0.1 mg/kg of cyclosporin.	1997	[56]
	Not reported	5	50 to 227 ng/mL	All infants had levels below the detection limit of 30 ng/mL. Breastfed infants of mothers on cyclosporine received less than 300 μg/day, with absorption amounts being undetectable. No nephrotoxic effects or other side effects were observed.	1998	[57]
		1	25 to 120 μg/L			
		1	87 to 440 μg/L			
	300 mg twice daily	1	79 to 286 μg/L on three separate occasions over a 10-week period	The breast milk/maternal blood level ratio was 84%, but the infant had undetectable levels. The infant grew and developed normally.	2001	[58]
	5.3 mg/kg per day	1	403 μg/L	The estimated dose the infant would ingest through breast milk was 0.06 mg/kg/day (1.1% of the weight-adjusted maternal dose), 1% of the therapeutic dose on a weight basis.	2003	[59]
	225 mg/day	1	465 μg/L in foremilk and 564 μg/L in hindmilk	The estimated dose the infant would ingest through breast milk was 0.08 mg/kg/day (2.1% of the weight-adjusted maternal dose). The cyclosporine concentration in the infant’s blood was below the detection limit of 25 μg/L.		
	250 mg/day	1	97.6 μg/L	The estimated dose the infant would ingest through breast milk was 0.01 mg/kg per day (0.2% of the weight-adjusted maternal dose). Cyclosporine levels in the infant’s blood were below the detection limit.		
	250 mg/day	1	117.7 μg/L ranged from 75 to 150 μg/L	The infant received a dose of 0.4% of the weight-adjusted maternal dose. The infant’s blood concentration was below the detection limit of 25 μg/L.		
	150 mg twice daily	1	Two points measurement on day 8 yielded 84 and 144 μg/L	Infant exposure is estimated to be 0.5% of the maternal weight-adjusted dose. Breastfeeding continued without any adverse effects observed.		
	100 mg in the morning and 75 mg in the evening	1	46 μg/L	The estimated dose the infant would ingest through breast milk was 0.007 mg/kg, or 0.33% of the weight-adjusted maternal dose. The infant’s blood levels were undetectable (<10 μg/L), and no apparent clinical adverse effects from cyclosporine were observed.	2011	[60]
	200 mg/day	1	Not reported	The infant’s serum cyclosporine level was undetectable (with an assay lower limit of 15 mg/L). The mother continued breastfeeding for 5 months, during which her infant remained healthy and had normal renal function.	2011	[61]
	120 mg/day	1		The infant’s serum cyclosporine level was undetectable, with a lower assay limit of 15 mg/L.		
	5 mg/kg	1	Not reported	The infant’s serum cyclosporine concentrations after the morning feed were consistently undetectable (<30 μg/L).	2011	[62]
	200 mg/day	1	128 μg/L, 200 μg/L, and 364 μg/L on days 10, 30, and 50 after morning dose: on day 40, before her morning dose, was 207 μg/L	By 12 months of age, the infant was developing normally and showed no noticeable adverse effects from the drug in the breast milk.	2014	[63]
	1.5 mg/kg/day	1	15.5 μg/L in colostrum	In the newborn, cyclosporine disappeared within 2 days. No immediate complications were observed with this pregnancy.	2016	[64]
	200 mg/day	7	22.4 μg/L	The mean cyclosporine concentration in the colostrum was 22.40 ± 9.43 μg/L, with an estimated mean daily dose of 1049.22 ± 397.41 ng/kg/24 h. The average daily infant dosage was estimated to be 1.05 μg/kg.	2020	[65]
	125 mg in the morning and 100 mg at night, totaling 3 mg/kg/day	1	0.443 μg/L to 5.3 μg/L	At the three-month follow-up, both twin infants were growing and developing normally, with no adverse effects observed.	2022	[66]
Tacrolimus	3 mg/day	1	0.57 μg//L one hour after the dose	The average amount of tacrolimus that neonates would ingest through maternal milk was 151.4 ng/kg/24 h. The peak tacrolimus concentration in colostrum was observed 8 h after an oral dose, reaching 3.219 ng/mL. The low concentrations of tacrolimus in colostrum indicate that the neonates would ingest only trace amounts of the drug. The infant was developing normally, both physically and neurologically.	2003	[67]
	2 mg twice daily	1	Average 1.8 μg/L, with a milk-to-blood ratio of 0.23	The baby ingested about 0.5% of the maternal weight-adjusted dose. The authors calculated that an exclusively breastfed infant would receive a daily dose of 0.27 μg/kg, which is approximately 0.5% of the maternal weight-adjusted dose and less than 0.2% of the pediatric dose for organ transplant rejection.	2006	[68]
	Not reported	6	0.3 to 1.9 μg/L, with average 1.7 μg/L	Normal prenatal growth for the gestational age and postnatal growth for the infant’s postpartum age.	2010	[69]
	9.6 mg/day (range from 4.5 to 15 mg/day)	4	Not reported	Whole-blood drug concentrations between day 15 and day 27 after delivery were undetectable, with a lower limit of detection of <1.9 μg/L.	2012	[70]
	6 mg twice daily	1	Not reported	Infant’s drug blood level was less than 1 μg/L.	2012	[71]
	Dose not specified, but assumed to be 6 mg/day	14	0.8 μg//L average (range 0.1 to 1.6 μg//L)	All infants experienced a 15% daily decline in tacrolimus levels. The maximum estimated absorption from breast milk was 0.23% of the maternal dose (weight-adjusted). The highest dosage an exclusively breastfed infant would receive is 0.56 μg/day, which is equivalent to 0.23% of the maternal weight-adjusted dose.	2013	[72]
	Average 7.5 mg/day	8	Average 0.93 ng/mL	Infants were exposed to less than 0.3% of the mother’s weight-adjusted tacrolimus dose through breast milk. With such low levels of neonatal drug exposure, it is considered unlikely to pose any health risk to the breastfeeding infant.	2013	[73]
	4 to 14 mg/day	14	Average 3.2 μg/L	The average amount of tacrolimus ingested by neonates through maternal milk was 151.4 ng/kg/24 h. The highest concentration was observed 8 h after an oral dose, reaching 3.219 ng/mL. The low concentrations of tacrolimus indicate that neonates are exposed to only trace amounts of the drug.	2013	[74]
	3 mg daily	2	Not reported	At 1 hour after breastfeeding, the first infant’s blood drug level was 0.2 μg/L at 10 days of age, while the second infant’s level was 0.5 μg/L at 7 days of age.	2014	[75]
	3.2 mg/day (range 2 to 5.5 mg)	13	The median levels were 3 μg/L at trough, 3.8 μg/L at 2 h, and 3.7 μg/L at 12 h	The relative infant dose in breastfed infants was less than 1%, and the drug levels in the infant’s blood were below detectable limits.	2018	[76]
	1.5 mg twice daily	1	At 4 days postpartum, the milk level was 1.1 μg/L; at 21 days postpartum, the milk levels were 1.4 μg/L at the time of the morning dose, 1.3 μg/L 4 h after the dose, 1.6 μg/L 8 h after the dose, and 1.4 μg/L 12 h after the dose	The breast milk-to-maternal blood ratio ranged from 0.40 to 0.64. Tacrolimus was undetectable in the neonate three weeks after birth. The authors estimated that a fully breastfed infant would receive 0.4% of the mother’s weight-adjusted dose.	2021	[77]
	Not reported	1	0.5 μg/L	The infant showed normal weight gain and motor development, with no indications of metabolic disorders or significant infections. The child’s exposure to the drug was extremely low, with the blood concentration approximately 90 times lower than the mother’s.	2024	[78]
Everolimus	2 mg daily during pregnancy	1	Undetectable levels (<0.5 μg/L) in colostrum 1 day postpartum	Estimated elimination half-life of everolimus was estimated at 86 h in the newborn.	2016	[64]
	0.5 mg/day	1	Highest level was 66 ng/L	The estimated infant dose of the drug was 4.224 ng/kg/24 h, which accounted for 0.38% of the mother’s dose.	2017	[79]
Azathioprine	75 mg/day	1	Peak colostrum levels (2 days postpartum) 2 and 8 h after oral dose, being 3.4 and 4.5 μg/L, respectively	The milk levels in these two mothers were equivalent to 0.05% and 0.6% of the maternal weight-adjusted doses, respectively. Infant serum levels were not assessed.	1982	[80]
	25 mg oral dose	1	Peak 6-MP milk level of 18 μg/L occurred 2 h after oral dose (7 days postpartum)			
	Not reported	2	Not reported	The infants exhibited normal blood cell counts, no increase in infections, and an above-average growth rate.	2008	[81]
	100 mg/day	1	6-MP was not detected five weeks after birth	With a detection limit of 5 μg/L, the infant would have ingested a maximum of 0.09% of the mother’s weight-adjusted dose. The child remained healthy and breastfed for 12 months.	1995	[55]
	1.2 to 2.1 mg/kg/day	4	Not reported	At 3 to 3.5 months of age, none of the infants had detectable blood levels of 6-TGNs and 6-MP.	2006	[82]
	100 mg/day	2	In 5 and 6 milk samples of each subject collected over a 24 h period, 6-MP was undetectable (<5 μg/L)	The absolute relative infant dose would have been under 0.09% of the maternal weight-adjusted dose, and no adverse effects were observed in the infants.	2006	[83]
	75 mg/day	1	Not reported	At the 1-month follow-up, the growth and development of the breastfeeding infant were reported as normal.		
	50 mg/day	1	Not reported	No adverse effects were observed in this child during the 2-month follow-up.		
	75 to 150 mg/day	10	Only one woman on 100 mg/day of azathioprine had detectable 6-MP in her milk; on day 28 postpartum, milk concentrations were 1.2 μg/L at 3 h and 7.6 μg/L at 6 h after the dose; no 6-MP was found in any of the other 29 milk samples	6-MP and 6-TGN were undetectable in the neonatal blood. None of the ten neonates showed clinical or hematological signs of immunosuppression during the first 28 days postpartum. One infant had a slightly low neutrophil count, but the overall white cell count remained normal.	2007	[84]
	75–200 mg/day	8	Peak 6-MP concentrations in milk were observed within the first 4 h after the dose, ranging from 2 to 50 μg/L	The estimated infant intake was less than 0.008 mg/kg body weight per 24 h, representing less than 1% of the maternal weight-adjusted dose.	2008	[85]
	100 mg (1.4 mg/kg) daily	1	Not reported	At both 8-days and 3-months postpartum, 6-TGNs were undetectable in the infant’s blood. Over the 6-month follow-up period, the child thrived and experienced no infections.	2009	[86]
	Median dose 150 mg/day (range 100 to 250 mg/day)	11	Not reported	There were no differences in mental or physical development between the two groups of infants, nor was there any variation in the incidence of other infections between the groups.	2011	[87]
	1.93 mg/Kg (AZA), 0.94–1.32 mg/Kg (6-MP)	Mothers taking either azathioprine (n = 28) or 6-MP (n = 2)	Not reported	In this cohort, nine infants were breastfed for an average of 7 months (ranging from 3 to 13 months). No statistically significant differences were observed between breastfed and formula-fed infants across any of the 12 survey domains.	2013	[88]
Prednisone	Single 10 mg oral dose	1	28.3 μg/L	N.A.	1975	[89]
	Single 20 mg oral dose	1	102 μg/L	N.A.	1981	[90]
	10 to 80 mg/day	6	Milk concentrations were 5% to 25% of those in serum.	At a daily dose of 80 mg of prednisolone, the infant would consume less than 0.1% of that dose, which is equivalent to less than 10% of the infant’s natural cortisol production.	1985	[91]
	Single 50 mg (intravenous dose)	3	Only 0.025% of the prednisolone dose (ranging from 0.010% to 0.049%) was found in the milk	N.A.	1993	[92]
	2 mg every 12 h	1	Prednisone levels in milk were undetectable (<4 μg/L) after 12 h, while prednisolone levels were undetectable after 6 h	The weight-adjusted infant dosages were 0.58% and 0.35% of the maternal prednisone dose and 0.18% and 0.09% of the maternal prednisolone dose.	2019	[93]
	15 mg every 24 h	1				
Belatacept	10 mg/kg monthly	1	Not reported	Normal growth and cognitive development.	2020	[94]
	Not reported	5	Not reported	No reports of problems breastfeeding or issues in the children.	2023	[95]

(6-MP: 6-mercaptopurine; 6-TGNs: 6-thioguanine nucleosides; AZA: azathioprine; N.A.: non-applicable).

### 4.3. mTOR Inhibitors

Mammalian target of rapamycin (mTOR) inhibitors are a class of drugs that disrupt the complex mTOR signaling pathway, thereby reducing T-cell growth and the alloimmune reaction. Two mTOR inhibitors have been approved and are used in kidney transplantation: sirolimus and everolimus. These drugs interact with immunophillins needed to inhibit cell growth and proliferation (Table 1). The primary trigger for the proliferation of activated T cells in this instance is IL-2. The IL-2 receptor is activated by IL-2 produced in an autocrine manner, which propagates the proliferation signal through PIK3 and Akt pathways. The inhibition of mTOR interferes with the metabolic status and differentiation of activated T cells, limiting their proliferation [96] (Figure 1). Because of the nephrotoxic effects associated with calcineurin inhibitors, mTOR inhibitors have emerged as immunosuppressive agents for use after transplantation.

#### 4.3.1. Sirolimus

Sirolimus has a high molecular weight (914.2), which may limit its transfer into breast milk, along with its low oral bioavailability. However, it has a long half-life of around 60 h in adults, and prescribing information notes that only trace amounts were detected in the milk of lactating rats [97].

Information regarding breastfeeding in human patients taking sirolimus is scarce, with only one case report available about a kidney–pancreas recipient. Unfortunately, this report lacks any details about the levels of the medication in both maternal and infant plasma, as well as in breast milk [72]. Therefore, further research on this drug is needed to begin analyzing its compatibility with breastfeeding.

#### 4.3.2. Everolimus

Similar to sirolimus, everolimus has a high molecular weight of 958 and a low oral bioavailability. While its half-life of 30 h is relatively long, it is shorter than that of sirolimus [97]. Currently, two case reports have examined everolimus levels in the breast milk of transplant recipients after childbirth, although neither patient elected to breastfeed. In one of them, a female transplanted patient received everolimus (2 mg/day) during pregnancy and postpartum. Although she did not breastfeed, colostrum collected one day after delivery showed undetectable everolimus levels. Serial plasma measurements indicated an estimated everolimus elimination half-life of 86 h in the newborn [64]. The second study on a transplant recipient receiving everolimus (0.5 mg) during pregnancy and postpartum reported a pre-dose everolimus level of 33 ng/L, peaking at 66 ng/L four hours post-dose. Levels at 2, 6, 8, and 12 h post-dose ranged from 45 to 51 ng/L, and the estimated dose for infants was calculated to be 4.22 ng/kg/24 h, representing 0.38% of the dose given to the mother [79] (Table 2).

### 4.4. Corticosteroids

Corticosteroids encompass both the primary endogenous glucocorticoid, cortisol, and synthetic therapeutic agents like prednisone and methylprednisolone. In transplantation medicine, glucocorticoids are the most commonly used corticosteroids due to their immunomodulatory properties. These compounds exert their effects by binding to the glucocorticoid receptor, forming a complex that regulates gene expression. This complex enhances the production of anti-inflammatory proteins within the nucleus (transactivation) while simultaneously suppressing pro-inflammatory protein expression in the cytosol by preventing the migration of transcription factors into the nucleus (transrepression) [28] (Figure 1).

No negative effects have been observed in breastfeeding infants when mothers use any corticosteroid while nursing. While it is frequently advised to refrain from breastfeeding for four hours after taking a dose, this practice might be unnecessary as the levels of prednisone in breast milk are quite low [98]. Most commonly used corticosteroid in kidney transplant regimens is prednisone.

The reports found on the use of prednisone during breastfeeding can be seen in Table 2. These articles’ study patient groups ranged from 1 to 6 individuals, and the levels of prednisone found in breastmilk ranged from undetectable (<4 μg/L) to 102 μg/L. Only 2 of these studies reported levels of prednisone in the infant’s blood, and the weight-adjusted dosages were between 0.1 and 0.58%, which is, in principle, considered safe. Table 2 shows a summary of these studies.

The use of steroids during pregnancy carries the risk of developing steroid-induced diabetes mellitus, characterized by an unusual rise in blood sugar levels linked to the administration of glucocorticoids, which may require careful monitoring and management to minimize potential complications for both the mother and the baby. Although limited documentation exists on this topic, this factor should be taken into account when evaluating the choices regarding corticosteroid therapy.

### 4.5. Belatacept

As an alternative to more traditional treatments, to prevent rejection and avoid their long-term side effects, new drugs to block T-cell activation have been recently developed. Belatacept is a protein resulting from combining the Fc region of immunoglobulin IgG1 and the extracellular domain of CTLA-4, therefore termed (CTLA-4)-Ig. To activate a T cell and elicit an immune response, the antigen-presenting cell must provide two types of signals to the T cell. One involves the major histocompatibility complex (MHC) in conjunction with the antigen, while the other signal is provided by the CD80 or CD86 molecule. Belatacept interacts the CD80 and CD86 molecules, resulting in the inhibition of the second signal from CD28 [99] (Figure 1). Belatacept has been the last immunosuppressive drug to be approved by American and European agencies since 2012 [100,101] and has shown significant improvement in renal function following kidney transplantation, as compared to CNIs [102]. Due to its recent approval, the availability of studies on belatacept and breastfeeding are limited to two studies, in which transplanted mothers breastfed their babies while maintaining the belatacept treatment, with normal growth and cognitive infant development reported [94,95] (Table 2).

The long-term management of maintenance immunosuppression in kidney transplant recipients is still complicated. While CNIs remain the preferred treatment option, their nephrotoxicity necessitates continuous evaluation and the search for alternatives. Current options include belatacept or mTOR inhibitors. While treatment with belatacept results in better kidney function at the 7-year evaluation compared to CNIs, it presents high initial rejection rates.

## 5. Additional Considerations

In addition to the immunosuppressive therapies discussed in this text, kidney transplantation often requires additional treatments to ensure compatibility with the graft and the patient’s survival. Consequently, prior to deciding about breastfeeding, it is crucial to assess the compatibility of these substances. Some examples are antihypertensive medication such as Angiotensin-Converting Enzyme Inhibitors (ACEIs)/Angiotensin II Receptor Blockers (ARBs), Calcium Channel Blockers (CCBs), and Sodium–Glucose Transport Protein 2 Inhibitors (SGLT2i); lipid-lowering agents like statins, antibiotics, and Erythropoiesis-Stimulating Agents (ESAs); and gastrointestinal protective drugs such as proton pump inhibitors (PPIs) or H2 blockers. A promising option is the combination of a low dose of tacrolimus with an Angiotensin-Converting Enzyme Inhibitor (ACEi)/Angiotensin II Receptor 1 Blocker (ARB) treatment to achieve adequate immunosuppression and protection against chronic scarring [103]. However, the compatibility of these agents with breastfeeding should be clinically evaluated within the context of the patient’s overall treatment plan.

The immunosuppressive treatments explained in this review focus on TCMR; however, AMR is a major contributor to late graft loss in kidney transplant recipients. Patients with high levels of donor-specific antibodies are at a higher risk of AMR and, consequently, graft loss [104]. Plasmapheresis, also known as plasma exchange, is the primary treatment used in kidney transplantation to remove donor-specific antibodies. Regarding its compatibility with lactation, most of the available evidence comes from plasma-exchange treatments during pregnancy in thrombotic microangiopathies, lipid disorders, and a variety of autoimmune diseases [105]. The use of plasmapheresis during pregnancy is primarily based on individual case reports, as there is a lack of high-quality studies and conclusive evidence-based guidelines. Therefore, scientific evidence on the compatibility of plasma-exchange therapy with breastfeeding in kidney transplantation is too limited to be properly assessed.

## 6. Discussion

In addition to the decrease in fertility rates, women who have undergone transplantation face an increased risk of preterm births, while babies born to these mothers often have low birth weights and may experience intrauterine growth restriction [2,3]. Given the established significance of breastfeeding in these situations, it is crucial to ensure that these infants have access to maternal milk. Due to the traditional discouragement of breastfeeding among transplanted mothers, there is limited information on the compatibility of certain immunosuppressant medications with lactation. Nonetheless, the latest guidelines increasingly highlight the compatibility of post-transplant treatment with breastfeeding, drafting a promising and optimistic outlook.

In 2013, Thiagarajan et al. released guidelines concerning breastfeeding after transplantation, organized around three specific scenarios. First, breastfeeding should be avoided when medications with known harmful effects are being used. Second, breastfeeding might either continue or be temporarily halted depending on the newborn’s serum levels of drugs that are typically regarded as safe at low concentrations, like cyclosporine, tacrolimus, corticosteroids, and azathioprine. Third, careful consideration is necessary when using medications with uncertain safety profiles, including mTOR inhibitors. These guidelines highlighted the need to find a balance between the health outcomes of the mother and infant while managing immunosuppressive therapies after transplantation during lactation [44]. Although more than a decade has passed since these recommendations, information regarding the levels of mTOR inhibitors in breast milk is still limited, underscoring the necessity for conducting further research on this topic. Furthermore, there appears to be a level of inconsistency in the existing studies concerning the presence of post-transplant immunosuppressive medications in breast milk, likely due to the unclear timing of sample collection in relation to maternal dosing and variations in assay methods.

Earlier pediatric guidelines have stated that some medications are present in human milk only in minimal amounts, and their mere presence does not automatically imply a risk to the infant. As a result, healthcare professionals are encouraged to consider multiple factors when providing guidance to those who require medication during breastfeeding [51].

Although there are currently reports with data on the subject, the compatibility of immunosuppressive drugs with lactation remains an area of limited research. The available reports are scarce and involve small patient cohorts, making it difficult to draw definitive conclusions. While low drug transfer to breast milk and minimal infant exposure have been reported, there is significant variability among studies. Additionally, follow-up periods are often short, ranging from 1 to 3 months in most cases, with only a few extending to 12–18 months. This limited timeframe restricts the ability to assess potential long-term effects in infants. Despite the theoretical safety suggested by low drug levels in breast milk, the absence of robust, long-term data prevents definitive recommendations for now. More extensive, well-designed studies with larger sample sizes and longer follow-up are necessary to evaluate the true impact of immunosuppressive therapy during lactation. Until then, the decision to breastfeed while on these medications should be individualized, weighing potential benefits against unknown risks.

When seeking the optimal approach to reconcile the use of immunosuppressant medications with lactation, adjusting the timing of drug administration and breastfeeding may be crucial. This can help minimize the exposure of the infant to the medications while ensuring the mother’s health is maintained. Strategies such as coordinating the timing of medication doses to occur immediately after a breastfeeding session or during periods when the infant will not be nursing for a longer time could be key to achieving both effective treatment and safe breastfeeding.

## 7. Conclusions and Future Directions

Based on the currently available evidence, it can be concluded that the indications suggest that breastfeeding may be compatible with the use of the studied medications. However, several limitations must be considered: the small sample sizes, the lack of methodological consistency across studies, and the absence of long-term follow-up. Furthermore, to aid in drawing conclusions about each medication, this review has examined them separately. Nevertheless, treatments following a kidney transplant generally consist of combinations of them.

Regarding the need to generate more consistent and reliable scientific evidence, it is essential to design prospective studies with matched control groups and, if possible, multicenter collaborations. Sample analysis should be scheduled from the first month (short term), continuing through 6–18 months (mid-term), and ideally extending follow-up to at least 5 years (long term). This framework is crucial to effectively monitor cognitive and growth development, immune response, allergy development, as well as metabolic and neurological health in infants exposed to maternal medication through breastfeeding.

Future clinical guidelines should focus on maintaining the therapeutic effect in the mother while minimizing drug exposure in the infant. To achieve this, several factors need to be taken into account: the dosage and frequency of the treatment, its pharmacokinetics, the infant’s health status, and the implementation of a breastfeeding schedule that maximizes the time interval between medication intake and nursing sessions. Overall, the prevailing perspective is that adjusting immunosuppressive dosages, in conjunction with oversight from a high-risk obstetrician, is crucial for ensuring safe breastfeeding [106].

In conclusion, while significant progress has been made in recent decades, much work remains to be conducted. The continuous updating of best practices and guidelines, incorporating the latest research on the topic, is essential for providing optimal care. Achieving a balance between the health benefits for both the mother and child while minimizing potential risks to the infant is key to advancing this area of clinical practice.

## Figures and Tables

**Table 1:** Most commonly used immunosuppressive drugs.

Group		Drug	Blocking Mechanisms
Immunophilin-binding treatments	Calcineurin inhibitors (CNIs)	Ciclosporin	Binds to cyclophilin, forming a complex that inhibits calcineurin, resulting in reduced cytokine production and diminished T-cell proliferation
		Tacrolimus	Binds to FK506-binding protein 12, forming a complex that inhibits calcineurin, thereby reducing cytokine production and T-cell proliferation
	mTOR inhibitors	Everolimus	Bind to FK506-binding protein 12, which, in turn, hinders mTOR, leading to a reduction in cytokine-induced T-cell proliferation
		Sirolimus	
Co-stimulation blockers	Cytotoxic T-lymphocyte-associated protein 4 (CTLA4)-Ig	Belatacept	Blocks co-stimulation of T-cell activity by CD28
Antimetabolites	Thiopurine	Azathioprine	Blocks purine production, leading to a decrease in T-cell growth
Corticoids	Glucocorticoids	Prednisone	Decrease the levels of circulating lymphocytes, monocytes, and eosinophils and suppress the production of cytokines
		Prednisolone	

## Abbreviations

The following abbreviations are used in this manuscript:

ESRD	End-stage renal disease
NTPR	National Transplant Registry
AMR	Antibody-mediated rejection
TCMR	T-cell mediated rejection
M/P	Milk-to-plasma concentration ratio of drugs
RID	Relative infant dose
CNIs	Calcineurin inhibitors
6-MP	6-mercaptopurine
6-TG	6-thioguanine
mTOR	mammalian target Of rapamycin
